# More than kin, less than kind: one family and the many faces of diabetes in youth

**DOI:** 10.1590/2359-3997000000312

**Published:** 2017-12-01

**Authors:** Luciana F. Franco, Renata Peixoto-Barbosa, Renata P. Dotto, José Gilberto H. Vieira, Magnus R. Dias-da-Silva, Luiz Carlos F. Reis, Fernando M. A. Giuffrida, Andre F. Reis

**Affiliations:** 1 Universidade Federal de São Paulo São Paulo SP Brasil Disciplina de Endocrinologia, Universidade Federal de São Paulo (Unifesp), São Paulo, SP Brasil; 2 Universidade do Estado da Bahia Departamento de Ciências da Vida Salvador BA Brasil Departamento de Ciências da Vida, Universidade do Estado da Bahia (UNEB), Salvador, BA, Brasil

## Abstract

Identification of the correct etiology of diabetes brings important implications for clinical management. In this report, we describe a case of a 4-year old asymptomatic girl with diabetes since age 2, along with several individuals in her family with different etiologies for hyperglycemia identified in youth. Genetic analyses were made by Sanger sequencing, laboratory measurements included HbA1c, lipid profile, fasting C-peptide, pancreatic auto-antibodies (glutamic acid decarboxylase [GAD], Islet Antigen 2 [IA-2], and anti-insulin). We found a Gly178Ala substitution in exon 5 of *GCK* gene in three individuals co-segregating with diabetes, and type 1 diabetes was identified in two other individuals based on clinical and laboratory data. One individual with previous gestational diabetes and other with prediabetes were also described. We discuss difficulties in defining etiology of hyperglycemia in youth in clinical practice, especially monogenic forms of diabetes, in spite of the availability of several genetic, laboratory, and clinical tools.

## INTRODUCTION

Identification of the exact etiology of diabetes brings important implications for clinical management, therapeutic choice, medical follow-up, screening of clinical complications, and prognosis, as well as genetic counseling when applicable.

Currently, in addition to more common forms such as type 1 and type 2 diabetes, molecular diagnosis of monogenic forms of diabetes has gained momentum due to the ever growing availability of centers performing genetic testing. In this report, we describe a family in whom several different types of diabetes have been diagnosed, underscoring the caveats of correctly identifying the etiology of diabetes.

## FAMILY PRESENTATION

**Figure 1:**
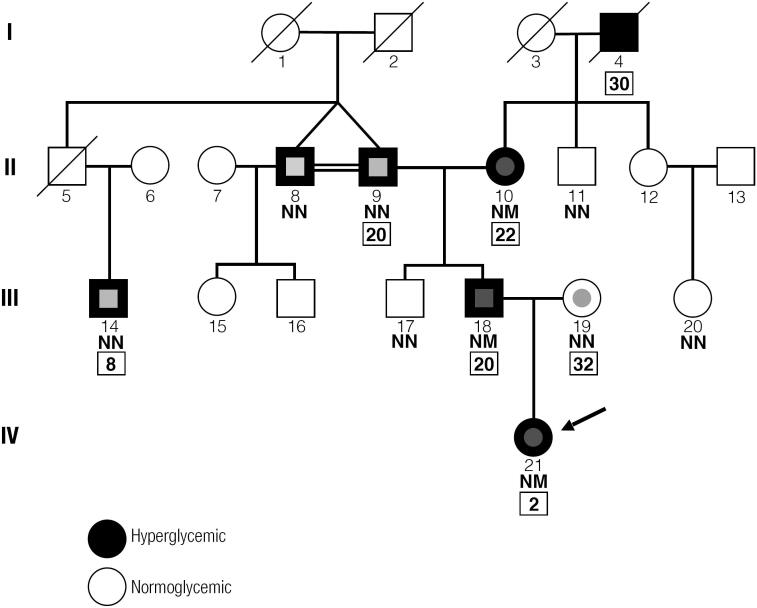
Pedigree of the studied family. Hyperglycemic and normoglycemic individuals depicted as black and white circles/squares, respectively. NN: normal-normal; NM: normal-mutated; regarding *GCK* Gly178Ala substitution. Normal type numbers refers to consecutive patient numbering as described in the text. Bold type numbers enclosed in borders describe age at diagnosis. The arrow indicates the proband. Individuals 10, 18, and 21 are GCK-MODY; individuals 9 and 14 have type 1 diabetes; individuals 8 has prediabetes; individual 19 has previous gestational diabetes.

The proband is a 4-year old asymptomatic girl (subject 21 on the pedigree chart, Figure 1), who was diagnosed with hyperglycemia in a routine visit with a pediatrician (fasting plasma glucose [FPG] 6.43 mmol/L) when she was 2 years old. Her current weight is 17 kg and height is 98 cm. She was a full-term newborn (birth weight 2450 g) with healthy neuropsychomotor development. Further glucose testing showed FPG 6.21 mmol/L, hemoglobin A1c (HbA1c) 6%, C-peptide 0.67 ng/mL, and negative pancreatic autoantibodies (glutamic acid decarboxylase [GAD], Islet Antigen 2 [IA-2], and anti-insulin all below detection threshold for each assay). Her mother (subject 19) had had a previous history of gestational diabetes at age 30, identified at 25 weeks gestational age, with progression to normoglycemia after pregnancy. Her father (subject 18) has shown hyperglycemia since age 20 without any symptoms or medication use. Her paternal grandfather (subject 9) was diagnosed with diabetes at age 20 and has been on insulin treatment since then; his monozygotic twin (subject 8) presented with prediabetes years ago, without specific treatment. Also, on the paternal side of the family, there is a 41-year old second cousin (subject 14), who was diagnosed with diabetes at age 8, also in use of insulin. Her paternal grandmother (subjected 10) is 57 years old with a history of hyperglycemia since 22 years old, identified in a routine scan, as part of a pre-employment assessment. She is asymptomatic, in use of metformin. Table 1 summarizes the main clinical and laboratory characteristics of family members.

**Table 1:** Clinical characteristics of studied individuals

Subject	Possible/definite etiology	Gender	Age	Age at diagnosis of diabetes (years)	BMI (kg/m^2^)	Dyslipidemia	Hypertension	Fasting glucose (mmol/L)	HbA1c (% / mmol/mol)	fasting C-peptide (ng/mL)	Pancreatic autoantibodies	Total cholesterol (mmol/L)	LDL-cholesterol (mmol/L)	HDL-cholesterol (mmol/L)	triglycerides (mmol/L)	Creatinine (mg/dL)	treatment
7	Normoglycemic	F	67	NA	25	–	–	4.9	5.5 / 37	NT	NT	5.4	3.4	1.5	1.2	0.9	None
8	Prediabetes	M	66	NA	25.4	+	+	6.0	5.8 / 40	6.9	–	5.3	3.4	1.3	1.2	1.0	Diet
9	Type 1 diabetes	M	66	20	28.7	+	+	12.4	6.3 / 45	< 0.1	–	3.7	1.9	1.5	0.5	0.9	NPH + Lispro
10	GCK-MODY	F	57	22	23.8	–	–	6.4	6.6 / 49	1.1	–	6.2	4.1	1.8	0.5	0.6	Metformin
11	Normoglycemic	M	55	NA	28.1	+	+	4.9	5.7 / 39	NT	–	6.2	3.9	1.0	2.8	1.0	None
14	Type 1 diabetes	M	41	8	32.8	–	–	11.4	6.4 / 46	< 0.1	IA2 +	4.1	2.3	1.6	0.6	0.8	NPH + Lispro insulin
15	Normoglycemic	F	37	NA	21.1	–	–	4.2	4.8 / 29	NT	NT	3.9	1.9	1.8	0.4	0.7	None
16	Normoglycemic	M	35	NA	32.4	–	–	5.3	5.5 / 37	3.8	–	5.1	3.3	1.1	1.4	1.1	None
17	Normoglycemic	M	26	NA	26.9	–	–	5.1	6.1 / 36	NT	–	4.3	2.8	1.1	0.9	0.9	None
18	GCK-MODY	M	30	20	27.2	–	–	7.2	6.1 / 43	1.1	–	4.6	2.9	1.3	0.5	0.9	None
19	Previous GDM	F	34	NA	24.5	–	–	5.0	5.6 / 38	NT	–	5.6	3.8	1.4	0.9	0.7	None
20	Normoglycemic	F	28	NA	21.7	–	–	5.0	4.7 / 28	NT	NT	4.3	2.4	1.7	0.6	0.8	None
**21**	**GCK-MODY**	**F**	**4**	**2**	*	**-**	**-**	**6.2**	**6.0 / 42**	**0.67**	**-**	**4.0**	**2.4**	**1.1**	**1.1**	**0.6**	**Diet**

GDM: gestational diabetes mellitus; F: female; M: male; NA: not applicable; NT: not tested;

* BMI was not calculated due to age, please see text for weight and height data. Proband data (subject 21) in boldtype. To convert total cholesterol, HDL-cholesterol, and LDL-cholesterol to mg/dL, divide values by 0.0259; to convert triglycerides to mg/dL, divide values by 0.0113; to convert glucose to mg/dL, divide values by 0.0555.

The atypical clinical presentation of hyperglycemia in the proband, along with her family background and absence of immunologic markers specific for type 1 diabetes, raised the hypothesis of monogenic diabetes - more specifically Maturity-Onset Diabetes of the Young (MODY) caused by a glucokinase *(GCK)* mutation. Genetic testing showed a Gly178Ala substitution in exon 5. The same mutation was found in the proband's father (subject 18) and paternal grandmother (subject 10), and is depicted in Figure 1 as normal-mutated (NM) to demonstrate its heterozygous pattern. Her mother, paternal grandfather, grandfathers's twin brother, two second cousins, and normoglycemic uncles (subjects 8, 9, 11, 14, 17, 19, and 20) did not presented any *GCK* mutations and are depicted as normal-normal (NN). The clear co-segregation of the mutation with mild non-progressive hyperglycemia strongly suggests it to have a causal effect (Figure 1). This mutation has been described for the first time in medical literature in this family. It was reported briefly in a recent publication by the Brazilian Monogenic Diabetes Study Group (BRASMOD), a multicenter group of researchers in the field of Monogenic Diabetes (1), but personal clinical data are presented here for the first time.

## METHODS

*GCK, HNF1A,* and *HNF4A* genes were analyzed by Sanger sequencing, using previously described primers and parameters (2). Pathogenicity of mutations was assessed by the American College of Medical Genetics and Genomics (ACMG) guidelines (3). *HNF1B* genotyping was performed with previously described methods (4). Hemoglobin A1 c (HbA1 c) was analyzed by High-Performance Liquid Chromatography (HPLC). C-peptide was analyzed by chemiluminometric assay. GAD antibodies have been assessed by enzyme-linked immunosorbent assay (ELISA); IA-2 and anti-insulin autoantibodies were assessed by radioimmunoassay. Diabetes and prediabetes were diagnosed according to the American Diabetes Association criteria (5). Arterial hypertension was defined as systolic blood pressure (SBP) ≥ 140 mmHg and/or diastolic blood pressure (DBP) ≥ 90 mmHg, or current use of antihypertensive medication and history of hypertension (6). Subjects were considered to have dyslipidemia either if levels of LDL-cholesterol were ≥ 4.14 mmol/L, HDL-cholesterol ≤ 1.04 mmol/L, triglycerides > 2.26 mmol/L, or if they were using lipid lowering medications (statin/fibrates) (6). The remaining clinical and laboratory data were collected from each patient's medical records. All patients or relatives responsible for the patients have provided informed consent, with approval by Hospital São Paulo's Ethics Committee.

## DISCUSSION

In this article we have described a family with multiple types of diabetes/hyperglycemia, diagnosed in infancy or early adulthood, underscoring the issue of etiological definition of diabetes in this age group, commonly dealt with in clinical practice. As far as we know, this is the first Brazilian report with these features.

The correct differentiation of monogenic forms from type 1 diabetes has significant clinical impact in treating the two most important differential diagnoses. Some subtypes of monogenic diabetes can be treated with sulfonylureas, like those caused by mutations in genes such as *HNF1A* and *HNF4A* (7), whereas others such as GCK-MODY may not need medical treatment at all (8). This is in contrast with type 1 diabetes, in which insulin therapy is required. A correct definition of diabetes etiology is often challenging, despite the availability of biochemical tests, pancreatic autoantibodies assessment, and molecular biology tools. In this regard, Patel and cols., in the United Kingdom, have recently proposed a genetic score with 30 variants to stratify the risk of development of type 1 diabetes. The study has suggested that the genetic score has shown good discriminative power to distinguish type 1 diabetes from monogenic forms (9) and in another analysis, the score was also useful to differentiate young type 2 diabetes (10). A meta-analysis by the same group assessed which clinical parameters could be relevant in the etiological differential diagnosis of the many types of diabetes. Age at diagnosis (< 35 years) and the period for insulin requirement (until 6 months after diagnosis) seem to be useful in distinguishing between type 1 and type 2 diabetes (11).

In this context, the use of biochemical markers could also be important. C-peptide is produced in concentrations equimolar to insulin and thus correlates with pancreatic residual function. C-peptide is more helpful 3 to 5 years after diagnosis, when its persistence may indicate type 2 diabetes or monogenic diabetes. Besides, undetectable levels at anytime in disease progression suggest type 1 diabetes. Normal reference values of C-peptide are variable in literature, but fasting C-peptide < 0.6 ng/mL usually indicates marked impairment of beta-cell function (12). C-peptide can be measured either in fasting or after a stimulus (with a mixed meal or glucagon) to detect beta-cell residual function. Some authors showed that urinary C-peptide- creatinine ratio (UCPCR) is a useful alternative method to measure C-peptide that can discriminate *HNF1A/ HNF4A* MODY from long-duration type 1 diabetes, although this test is not routinely performed in our clinic (13). In the present family all GCK-MODY subjects presented fasting C-peptide above 0.6 ng/mL, while those with type 1 diabetes (either confirmed by detection of autoantibodies or not) showed undetectable values (Table 1), suggesting it as a good discriminator between both diabetes subtypes.

Current methods of genetic tests for MODY mutations are expensive and time consuming. Patients should be carefully selected by clinical criteria for testing, as this can significantly increase the positivity detection rate (14). In order to enhance accuracy in the recruitment of suspected cases of MODY, a team of researchers developed software based in specific clinical data that calculates the patient's probability of having a positive molecular test for MODY mutations. This software uses data such as age, insulin requirement within 6 months after diagnosis, and familial history of diabetes. Moving on to genetic testing is suggested when the score is greater than 25% (15). Of note, all patients with GCK-MODY in the present family had a score above this cutoff point (data not shown). Other strategies for patient selection and mutation screening have been proposed. Shepherd and cols., investigating the prevalence of monogenic diabetes in U.K. pediatric clinics in patients younger than 20 years old, used a systematic approach of biomarker screening with UCPCR, islet autoantibodies (GAD and IA-2), and targeted genetic testing. They found 2.5% of patients to have monogenic diabetes. Authors suggested that this biomarker screening algorithm could be a rational and practical approach to identifying pediatric patients who are most suitable for genetic testing (16).

Therefore, an attempt for establishing diagnosis or rendering a provisional etiology in each case is discussed as follows.

The proband (subject 21) had a typical clinical presentation of GCK-MODY with mild hyperglycemia and no symptoms, along with classic laboratory findings like detectable levels of C-peptide and absence of pancreatic auto-immunity. This raises the question of which parent she has inherited her mutation from. If on the one hand her mother (subject 19) had previous gestational diabetes, on the other hand, her father (subject 18) has also had mild non-progressive hyperglycemia for several years. Her father also showed a clinical pattern of GCK-MODY, being diagnosed at age 20 without clinical manifestations.

The proband's mother, previously normoglycemic, had had a history of gestational diabetes identified at 25 weeks of pregnancy. Her oral glucose tolerance test [OGTT] showed glucose values of 4.2, 9.2, and 9.1 mmol/L in fasting, at 1, and 2 hours, respectively (5), and she was managed only with diet. After delivery, blood glucose returned to normal levels as shown by a further OGTT. She was unaware of any other cases of diabetes in her family. The inherited mutation could have reflected on birthweight, partially explaining why macrosomia did not occur (17). Several studies show that weight of children born with *GCK* mutations is usually ~700g lower compared to siblings who did not inherited the mutation. This finding is more robust when the mother also shows hyperglycemia caused by a *GCK* mutation, but it could be extrapolated to the gestational diabetes context. This is possibly due to lower insulin secretion during intrauterine life (18). Her paternal grandmother (subject 10) had a typical presentation of GCK-MODY with mild non-progressive asymptomatic hyperglycemia, identified in routine pre-employment testing when she was 22. Genetic testing successfully confirmed the *GCK* mutation in the grandmother and ruled it out in the grandfather, in whom clinical features are more resemblant of other forms of hyperglycemia. Besides typical clinical GCK-MODY presentation and familial cosegregation, Gly178Ala mutation is Likely Pathogenic according to ACMG guidelines, presenting 2 Moderate Criteria and 3 Supporting Criteria (3). Besides, this variant has not been found in population databases of exome sequences (19).

While the proband's paternal grandfather (subject 9) has a history of diabetes since his early youth, his monozygotic twin brother (subject 8) shows a prediabetes pattern that could resemble GCK-MODY (see below). He could have inherited not only the GCK-MODY mutation but also genetic predisposition to type 1 diabetes, which would hypothetically result in a more severe clinical presentation prevailing, but this was not the case as demonstrated by negative genetic testing in him. Furthermore, he had a history of diabetes diagnosed at age 20, presenting with polydipsia, requiring insulin since diagnosis, and showing undetectable levels of C-peptide. Thus a diagnosis of type 1 diabetes is strongly suggested. Negative GAD and IA-2 antibodies could simply reflect long disease duration, with the reduction of antibody titers that is usually observed in type 1 diabetes. A more rare condition could be the presence of an *HNF1A* or even *HNF4A* mutation in subject 9 and his twin brother with prediabetes. HNF1A-MODY presents mostly as overt diabetes, a phenotype markedly different from GCK- MODY. Genetic testing for these MODY-subtypes has been carried out in subject 9 with negative results. Coexistence of different MODY subtypes *(HNF1A* and *GCK)* has been described in a family in whom clinical predominance of the most severe subtype, i.e. *HNF1A,* occurred. A targeted NGS panel with several MODY genes could be an important tool in such cases (20,21).

An interesting feature of subject 9 is the presence of persistent proteinuria and hematuria, but with a normal retinal examination, together with normal creatinine clearance (102 mL/min/m^2^) and normal urinary tract ultrasound. This finding could suggest etiologies other than diabetes for nephropathy (22). Type 2 diabetes could be considered, but insulin requirement being present since diagnosis undermines this hypothesis. His twin brother (subject 8) presents with prediabetes diagnosed both by fasting glucose and HbA1c several years ago (approximately 10 years). No pancreatic autoimmunity has been demonstrated. This individual (subject 8) could either have type 2 diabetes-related prediabetes or preclinical type 1 diabetes. The absence of autoimmunity balances the scale toward the first hypothesis. Like his brother he has also presented with hematuria for several years and persistent proteinuria (around 0.85 g/L), renal cysts at ultrasound, and stable creatinine clearance over the last few years (ranging from 90 to 110 mL/min). One hypothesis for explaining the etiology of hyperglycemia with renal alterations, especially renal cysts, could be an *HNF1B* mutation, which has also been tested with negative results, consequently excluding this MODY subtype also in his twin brother (4,23).

On the paternal grandfather's side of the family, there is a 41-year old second-cousin (subject 14) without diabetic complications and a typical type 1 diabetes presentation, i.e., diagnosis at age 8, insulin treatment since then, and undetectable C-peptide. DKA was seen upon diagnosis and other episodes occurred during his life as well. GAD65 autoantibodies were negative (< 0.5 UI/mL), but IA-2 was positive (1.2 U/mL; normal range < 0.8 U/mL), strongly suggesting type 1A diabetes, thus the most likely etiology in this individual is autoimmune.

## CONCLUSION

Abnormal glucose levels are the common element present in different diseases such as GCK-MODY, prediabetes, type 1 diabetes, and gestational diabetes, especially in children, teenagers, and young adults. As a consequence, it can be challenging to correctly define a precise diabetes etiology. Improvement in the understanding of diabetes depends on thorough analysis of clinical data, genetic testing, and laboratory findings. Despite all recent advancements in molecular diagnosis, the definition of a clinical screening model for monogenic diabetes is still a challenge that demands the systematic study of several families in different populations. In conclusion, the accurate molecular and clinical diagnosis has significant impact on clinical management of a multifaceted disease such as diabetes, especially when running on the same family.
